# Oropharyngeal symptoms without systemic reactions as a risk factor for food allergen intolerance: a longitudinal pediatric study

**DOI:** 10.1186/s13223-022-00675-1

**Published:** 2022-04-18

**Authors:** Masaaki Hamada, Keigo Saeki, Yoshihiko Sakurai

**Affiliations:** 1 Department of Pediatrics, Yao Municipal Hospital, 1-3-1 Ryuge-cho, Yao, Osaka 581-0069 Japan; 2 Department of Epidemiology, Nara Medical University School of Medicine, Kashihara, Japan; 3 Department of Legal Medicine, Nara Medical University School of Medicine, Kashihara, Japan

**Keywords:** Food allergy, Local reaction, Oral food challenge test, Oral immunotherapy, Oropharyngeal symptom, Tolerance

## Abstract

**Background:**

The determinants of tolerance to food allergens are not fully understood. We aimed to elucidate the longitudinal association between oropharyngeal symptoms without systemic reactions (OSw/oS) and tolerance to food allergens.

**Methods:**

We included all patients diagnosed with single food allergy to egg (n = 121), milk (n = 55), and wheat (n = 41) using the oral food challenge test (OFC) from 2014 to 2017. These patients received oral immunotherapy at home and/or in the hospital after diagnosis by OFC. We compared the incidence proportion of tolerance within 2 years by OSw/oS and other variables for 217 patients with food allergy. We defined OSw/oS as isolated symptoms of oropharyngeal discomfort that occurred after ingestion of a safe dose of the allergenic food determined by the OFC in the first 6 months.

**Results:**

Of the 217 patients (median age 37.5 months, male 64.5%), 53 developed OSw/oS (24.4%), and 151 (egg, 85 milk, 36 and wheat, 30) attained tolerance in 2 years. Patients without OSw/oS showed a significantly higher incidence of tolerance than those with the symptoms (crude hazard ratio [HR] 5.62, 95% confidence interval [CI] 3.58–8.82, *p* < 0.001). The association was consistently significant in the multivariable model (adjusted HR 9.50, 95% CI 5.25–17.20, *p* < 0.001) independent of other risk factors for intolerance, such as concomitant bronchial asthma (adjusted HR 3.33), history of anaphylaxis (adjusted HR 2.16), milk allergy (adjusted HR 2.02), and allergic symptoms with low dose OFC (adjusted HR 1.52).

**Conclusion:**

Our results suggest that OSw/oS may be a risk factor for intolerance to food allergens. To reveal a high risk of food allergen intolerance may help patients and their families as well as healthcare professionals prepare for the challenge of continuing oral immunotherapy.

## Introduction

Food allergy is a common condition in nearly 10% of infants and can cause life-threatening allergic symptoms [1]. Even without treatment, fifty percent or more of children with food allergies attain immune tolerance as they grow [1]. Tolerance in food allergy is defined as the antigen-specific suppression of cellular or humoral immune responses [2], and is clinically characterized by a persistent state of clinical non-reactivity to allergens [3]. For the remaining pediatric patients with persistent food allergy, oral immunotherapy (OIT) is one of the feasible treatment options used safely and effectively to reduce the sensitivity to food antigens [4].

The determinants of tolerance to food allergens are not fully understood. Previous studies have reported several risk factors that interfere with the achievement of tolerance to food allergens, including high specific IgE antibody titers against the food, anaphylactic symptoms, complications of other allergic diseases, and slow reduction of IgE antibody titers [1, 5–7]. Oropharyngeal symptoms such as itching and/or angioedema of the lips, tongue, palate, and throat, sometimes accompanied by stinging pain, often develop during OIT [8] but have been considered as minor local hypersensitivity reactions to food allergens [9]. However, based on our experience of patients who cannot continue OIT due to oropharyngeal symptoms alone or achieve tolerance, we hypothesized that oropharyngeal symptoms without systemic reactions (OSw/oS) may be a risk factor for failure to achieve tolerance. In our preliminary study involving patients with single or multiple food allergies, we observed the association of oropharyngeal symptoms with intolerance in patients with egg allergy [10]. The present study aimed to elucidate the longitudinal association between OSw/oS and tolerance to food allergens using multivariable analysis.

## Methods

### Study design

This prospective cohort study followed patients, who were diagnosed with single food allergy to hen’s egg, cow’s milk, or wheat, for 2 years after the baseline oral food challenge test (OFC) conducted in the Yao Municipal Hospital from 2014 to 2017, and the incidence proportion of tolerance by the presence or absence of OSw/oS was compared. Inclusion was limited to patients with single food allergy so as to eliminate the influence of other food allergens. The guardians of all patients provided written informed consent to participate in this study at the time of baseline OFC. This study was approved by the Yao Municipal Hospital Ethics Committee (YMH-012221-108).

### The OFC procedure

**Fig. 1:**
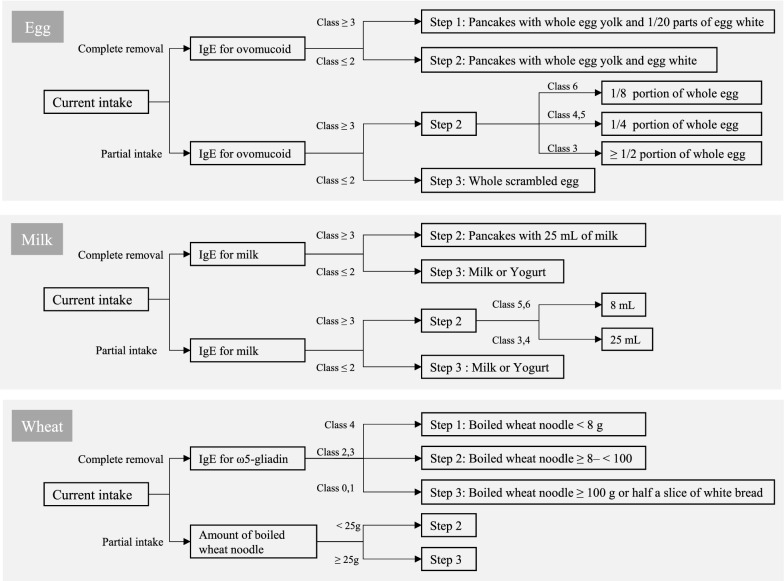
The procedure of oral food challenge tests (OFC). The dose of the baseline OFC was based on the extent of ingestion, specific IgE antibody titer, total IgE value, and the history of allergic symptoms. When the OFC was negative in step 1 or 2, the next step OFC was promptly performed. Conversely, the OFC was stepped down depending on the results of the first step 2 or 3 OFC. Preparation of foods used for the challenge is shown in Table 1

We conducted an OFC comprising three steps under direct medical supervision at our hospital. The challenging dose of the baseline OFC was determined by the current intake status (complete removal or partial intake) and specific IgE antibody titer (Fig. 1). The food items used for each step of the OFC are summarized in Table 1. The patients ingested 1/8, 3/8, and 1/2 of the total challenging dose at 30-min intervals, while a single dose was used for patients judged to be at high risk of severe allergic reactions based on history. When allergic symptoms occurred during the OFC, the patient stopped ingesting the suspected food and was diagnosed with food allergy. We determined the allergic symptoms in the OFC according to the Japanese guidelines for food allergy (Table 2) [11]. In addition, when any allergic symptom developed at low dose OFC (step 1 OFC for egg or wheat and step 2 OFC for milk), it was noted as “allergic symptoms with low dose OFC.” If the OFC was negative in steps 1 or 2, the next step of OFC was promptly performed.

**Table 1:** Food items used for the oral food challenge test

Food	Step 1	Step 2	Step 3
Egg	Pancakes with whole egg yolk and 1/20 parts of egg white	Pancakes with 1/8 whole egg for class 6 ovomucoid IgE Pancakes with 1/4 whole egg for classes 4–5 Pancakes with 1/2 or whole egg for class 3 or below	Whole scramble egg
Milk	Bread with 3 mL of milk (a slice of commercially available white bread)	Pancakes with 8 mL of milk for class 5 or more milk IgE Pancakes with 25 mL of milk for class 3–4 specific milk IgE	Raw milk or yogurt (100–200 mL) for class 2 or below milk IgE 25–50 mL of milk or yogurt in the case of transition from step 2
Wheat	Boiled udon (wheat noodles) less than 8 g	Boiled udon 8 g or more and less than 100 g	Boiled udon 100 g or more or half a piece of bread

The pancakes and scrambled egg were cooked by fully heating in a frying pan

The total dose at each step was reduced to 1/8–1/2 depending on the case

**Table 2:** Symptoms of food allergies

Organ	Symptoms
Skin	Erythema, urticaria, angioedema, pruritus, burning sensation, eczema
Mucous membranes	Conjunctival hyperemia and edema, pruritus, lacrimation, blepharedema Rhinorrhea, nasal congestion, sneezing Discomfort/swelling of the oral cavity/pharynx/lips/tongue
Respiratory system	Discomfort/itch/tightness in the pharyngolarynx, hoarseness, dysphagia, coughing, wheezing, retractive breathing, feeling of chest tightness, dyspnea, cyanosis
Gastrointestinal system	Nausea, vomiting, abdominal pain, diarrhea, hematochezia
Nervous system	Headache, lowered vigor, unrest, impaired consciousness, incontinence
Cardiovascular system	Decreased blood pressure, tachycardia, bradycardia, arrhythmia, coldness of limbs, pallor (peripheral circulatory failure)

### Follow-up of patients with dietary guidance according to OIT

We determined the safe dose based on both the final dose of the OFC and the grade of the allergic symptoms in the OFC according to a previous report [12]. During the 2-year follow-up from the baseline OFC, we instructed the guardians to allow the patients to ingest a safe dose of the allergenic food at home and report the number of intakes and occurrences of allergic symptoms at each regular outpatient visit. Based on the patient reports, we determined whether the patients could continue to ingest the food safely. Depending on the patients’ clinical course, an additional OFC was performed to confirm whether the maintenance dose was appropriate or whether the dose could be escalated. When the patients continuously ingested the allergenic food without major symptoms, we recommended a dose increase, although the final decision was based on their own and their guardians’ will. The guardians were instructed to allow the patients to ingest the allergenic food at least twice a week for those who did not increase the dose and at least thrice a week for those who increased the dose.

### Definition of tolerance

We set each allergen target dose as follows: a whole scrambled egg, 200 mL of milk, and 200 g of boiled wheat noodles (udon). Infants under 2 years of age received half of the target dose. Patients who reached the target dose within 24 months after baseline OFC were considered to have achieved clinical tolerance.

### Oropharyngeal symptoms without systemic reactions (OSw/oS)

OSw/oS was defined as discomfort in the oral cavity and pharynx without any other allergic reactions that occurred after ingesting a safe dose of the allergenic food within 6 months of the baseline OFC. Discomfort, such as spiciness, tingling, strangeness, itching, and numbness in the oral cavity and pharynx was essential for diagnosis. Although redness around the oral cavity may also be present, it was not a requirement for diagnosis of OSw/oS. We deliberately evaluated whether the patients’ symptoms and complaints, which were observed directly by doctor(s) during and after ingestion and/or were obtained from the careful interview with a patient and the guardian(s), corresponded to OSw/oS. Among infants who could not communicate verbally, doctor(s) and/or guardian(s) paid careful attention to facial expressions, as well as physical conditions, to avoid missing any changes that occurred after putting the food into the mouth. In fact, before ingesting the allergenic food, we confirmed that the patients consumed non-allergenic food without an unpleasant expression and without any reluctance. OSw/oS was recognized by unpleasant facial expressions that seemed different than the usual, with or without crying at ingestion.

### Measurement of other variables

We investigated the patients’ characteristics at the time of the baseline OFC, such as sex, age in months, concomitant atopic dermatitis, bronchial asthma and allergic rhinitis, history of anaphylaxis, allergic symptoms with low-dose OFC, total IgE antibody levels, and specific IgE antibody titers to food allergens. Specific IgE antibody titers to egg white, ovomucoid, milk, and wheat were determined using the Alastat 3G Allergy^®^ (Siemens Healthcare, Tokyo, Japan) and those to ω5 gliadin were determined using Immunocap^®^ (Thermo Fisher Scientific, Tokyo Japan).

### Statistical analysis

The time to achievement of tolerance to food allergens was analyzed using the Kaplan–Meier method. Cox proportional hazard model estimated the hazard ratio (HR) for intolerance and 95% confidence intervals (CI). The explanatory variables of the model included sex, age in months, concomitant bronchial asthma or allergic rhinitis, history of anaphylaxis, specific IgE antibody titer (egg white for egg allergy, milk for milk allergy, and wheat for wheat allergy), allergic symptoms with low-dose OFC (positive for step 1 or step 2 of OFC), allergen, and OSw/oS. Age in months and specific IgE antibody titers were converted to logarithms prior to hazard ratio analysis to reduce the skewness of distribution. All statistical analyses were performed using EZR ver.1.35 [13].

## Results

A total of 217 patients (median age at baseline OFC, 37.5 months; male, 64.5%) were included in the present study. These patients had egg allergy (n = 121), milk allergy (n = 55), and wheat allergy (n = 41). The proportion of patients who achieved tolerance within the observation period of 2 years was 70.2% for egg allergy (85/121), 65.5% for milk allergy (36/55), and 73.2% for wheat allergy (30/41) (Table 3). Of the 217 patients, OSw/oS was observed in 53 patients with egg or milk allergy (24.4%) but not in those with wheat allergy. The prevalence of OSw/oS was significantly higher in the intolerance group than in the tolerance group (56.1% vs. 10.6%, *p* < 0.001).

**Table 3:** Characteristics of 217 patients with positive oral food challenge test at baseline

Background factor	Tolerance group	Intolerance group	*p* Value
	n = 151	n = 66	
Egg allergy, n	85	36	
Male, n (%)	53 (62.4)	22 (57.9)	1.000
Age in months, median (IQR)	24 (13–32)	43 (24–64)	< 0.001
Atopic dermatitis, n (%)	73 (85.9)	32 (88.9)	0.775
Bronchial asthma, n (%)	7 (8.2)	11 (30.6)	< 0.001
Allergic rhinitis, n (%)	4 (4.7)	9 (25.0)	< 0.001
History of anaphylaxis, n (%)	22 (25.9)	18 (50.0)	0.012
Allergic symptoms with low dose OFC^a^	19 (22.4)	12 (33.3)	0.255
OSw/oS	14 (16.5)	32 (88.9)	< 0.001
IgE titer			
Total (IU/mL), median (IQR)	168.0 (50–363)	474.5 (266.5–1201)	< 0.001
Egg white (IUA/mL), median (IQR)	38.7 (19.7–79.9)	99.9 (64.9–208.8)	< 0.001
Ovomucoid (IUA/mL), median (IQR)	29.2 (11.6–56.8)	75.8 (43.6–157.8)	< 0.001
Milk allergy, n	36	19	
Male, n (%)	24 (66.7)	15 (71.4)	0.533
Age in months, median (IQR)	21 (17–25)	36 (25–50)	< 0.001
Atopic dermatitis, n (%)	31 (86.1)	18 (94.7)	0.653
Bronchial asthma, n (%)	3 (8.3)	12 (63.2)	< 0.001
Allergic rhinitis, n (%)	1 (2.8)	9 (47.4)	< 0.001
History of anaphylaxis, n (%)	14 (38.9)	15 (78.9)	< 0.001
Allergic symptoms with low dose OFC^a^	15 (41.7)	16 (84.2)	< 0.001
OSw/oS	2 (5.6)	5 (26.3)	0.041
IgE titer			
Total (IU/mL), median (IQR)	178.0 (58.5–477.5)	485.0 (252–1097)	< 0.001
Milk (IUA/mL), median (IQR)	22.9 (9.2–38.5)	83.3 (46.7–179.5)	< 0.001
Wheat allergy, n	30	11	
Male, n (%)	19 (61.5)	7 (63.6)	1.000
Age in months, median (IQR)	16 (13–27)	20 (18–26)	0.019
Atopic dermatitis, n (%)	26 (86.7)	10 (90.9)	1.000
Bronchial asthma, n (%)	6 (20.0)	9 (81.8)	< 0.001
Allergic rhinitis, n (%)	2 (6.7)	3 (27.3)	0.110
History of anaphylaxis, n (%)	4 (13.3)	10 (90.9)	< 0.001
Allergic symptoms with low dose OFC^a^	12 (40.0)	10 (90.9)	< 0.001
OSw/oS	0 (0.0)	0 (0.0)	1.000
IgE titer			
Total (IU/mL), median (IQR)	239.0 (127.5–437.8)	596.0 (193.5–1120)	0.115
Wheat (IUA/mL), median (IQR)	8.4 (2.5–18.9)	76.4 (13.5–195.0)	< 0.001
ω5 gliadin (UA/mL), median (IQR)	1.8 (0.8–5.1)	9.7 (4.3–29.5)	< 0.001

*n* number of patients, *IQR* interquartile range, *OFC* oral food challenge, *OSw/oS* Oral and pharyngeal symptoms without systemic reactions

^a^Low dose OFC: step 1 OFC for egg or wheat and step 2 OFC for milk

**Fig. 2:**
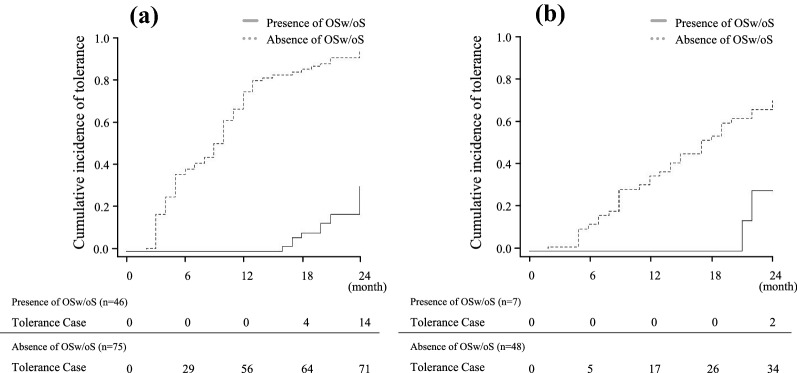
Kaplan–Meier survival curve showing tolerance rate of food allergies by the presence or absence of oropharyngeal symptoms without systemic reactions (OSw/oS). In wheat allergy, no OSw/oS were observed. **a** Egg allergy **b** Milk allergy

The patients having egg allergy and milk allergy without OSw/oS showed a significantly higher probability of achieving tolerance in 2 years than those with OSw/oS based on Kaplan–Meier analysis (*p* < 0.001 and *p* < 0.028, respectively, Fig. 2).

In the multivariable model, the prevalence of OSw/oS was significantly associated with intolerance (crude HR 5.62, 95% CI 3.58–8.82, *p* < 0.001; Table 4). The association between intolerance and OSw/oS remained significant (adjusted HR 9.50, 95% CI 5.25–17.20, *p* < 0.001), even after adjustment for potential confounders, such as sex, log transformed-age in months, bronchial asthma, allergic rhinitis, history of anaphylaxis, allergic symptoms with low-dose OFC, log transformed-specific IgE titers, and the type of food allergen (Table 4). Other than OSw/oS, the statistically significant independent risk factors for intolerance included concomitant bronchial asthma (adjusted HR 3.33, 95% CI 1.74–6.37), history of anaphylaxis (adjusted HR 2.16, 95% CI 1.47–3.17), milk allergy (adjusted HR 2.02, 95% CI 1.32–3.08), and allergic symptoms with low dose OFC (adjusted HR 1.52, 95% CI 1.01–2.28) (Table 4).

**Table 4:** Hazard ratio for food allergy tolerance during the 2-year follow-up

Explanatory variables	Crude HR (95% CI)	*p* Value	Adjusted HR (95% CI)^a^	*p* Value
Sex (male)	0.97 (0.70–1.33)	0.835	1.30 (0.91–1.80)	0.134
Age in month (log transformed)	0.17 (0.09–0.32)	< 0.001	1.02 (0.49–2.12)	0.959
Bronchial asthma	3.67 (2.17–6.20)	< 0.001	3.33 (1.74–6.37)	< 0.001
Allergic rhinitis	4.91 (2.33–10.30)	< 0.001	1.22 (0.52–2.87)	0.652
History of anaphylaxis	2.43 (1.75–3.37)	< 0.001	2.16 (1.47–3.17)	< 0.001
Allergic symptoms with low dose OFC^b^	2.40 (1.68–3.42)	< 0.001	1.52 (1.01–2.28)	0.043
OSw/oS	5.62 (3.58–8.82)	< 0.001	9.50 (5.25–17.20)	< 0.001
Specific IgE antibody titers (log transformed)^c^	0.49 (0.40–0.60)	< 0.001	0.75 (0.56–1.009)	0.058
Allergen				
Egg	0.97 (0.71–1.33)	0.865	1 (reference)	
Milk	1.30 (0.89–1.89)	0.169	2.02 (1.32–3.08)	0.001
Wheat	0.74 (0.49–1.10)	0.136	1.11 (0.68–1.83)	0.668

*HR* Hazard ratio, 95% *CI* 95% confidence interval, *OFC* oral food challenge, *OSw/oS* Oral and pharyngeal symptoms without systematic reaction

^a^Adjusted HR was estimated using a Cox proportional hazard model including all variables as dependent variables

^b^Low dose OFC: step 1 OFC for egg or wheat and step2 OFC for milk

^c^Specific IgE antibody titer is egg white, milk, and wheat, respectively

In the sensitivity analysis, when OSw/oS was confined to that occurring at the baseline (n = 37), the association between intolerance and OSw/oS at the baseline remained significant (adjusted HR 6.51, 95% CI 3.32–12.76, *p* < 0.001).

## Discussion

In the present study, 70% of the patients who underwent OIT for 2 years achieved tolerance to food allergens (egg, milk, and wheat) within the same time period. According to previous studies examining the natural history of food allergies, the rate of tolerance to egg allergy is 12% by the age of 6 years and 37% by the age of 10 years [14], that of milk allergy is 19% by the age of 4 years and 42% by the age of 8 years [15], while that of wheat allergy is 29% by the age of 4 years and 56% by the age of 8 years [16]. Considering these findings, the tolerance rate in our study was higher. We found that continuous intake of allergenic foods even without strictly planned OIT may help achieve tolerance, which is consistent with previous findings [17, 18].

We observed that OSw/oS had a significant association with the outcome of OIT after adjustment of egg, milk, and wheat allergens in the multivariable analysis, which further supports and expands our previous finding that patients with egg allergy and concomitant oropharyngeal symptoms have difficulty reaching tolerance [10].

OSw/oS has been considered a local hyperreaction to food allergens [8, 19] and has not been included in previously identified risk factors. Its association with tolerance to food allergens has attracted little attention [9]. To our knowledge, this is the first report on the association between OSw/oS and tolerance to allergenic foods. Consistent with some previous studies [5], we also demonstrated that patients with previously reported risk factors, including concomitant bronchial asthma and history of anaphylaxis at baseline, showed a significantly lower rate of achieving tolerance to food allergens. Even after adjusting for the effects of these risk factors using multivariable analysis, the association between OSw/oS and a lower rate of achieving tolerance was significant.

Although the mechanism by which OSw/oS interferes with tolerance to food allergens is unknown, some plausible mechanisms might explain this association. First, patients with OSw/oS may not be able to masticate allergenic food well due to oropharyngeal discomfort. Thus, food put into the mouth may move to the gastrointestinal tract with insufficient oral digestion, which, in effect, maintains the food allergen structure that can trigger an immune response. Consequently, it may be difficult to induce tolerance. However, because oral digestion in infants is naturally incomplete and develops along with teething and neural development, incomplete mastication is unlikely to be a major mechanism. Second, patients with OSw/oS may have allergic reactions in the gastrointestinal mucosa, similar to those in the oropharyngeal mucosa, but without clear local symptoms, such as abdominal discomfort and pain. Recent research has revealed that oral mucosa-associated immune system may affect the systemic immune system and may be involved in food allergy through the remodeling of the oral epithelial barrier [20], which may provide clues to elucidate the mechanism. Third, OSw/oS may simply reflect the severity of food allergy. Our results that the rates of history of anaphylaxis, the presence of bronchial asthma or allergic rhinitis, or IgE titers, as well as the occurrence of OSw/oS were higher in the intolerant group than in the tolerant group may account for the association between the presence of OSw/oS and the lack of tolerance. Furthermore, local differences in the propensity of mast cells to degranulate may be involved. However, OSw/oS was observed mainly in egg allergy, to a lesser extent in milk allergy, and not observed in wheat allergy. If the incidence of OSw/oS differs among food allergens, though a relatively small number of participants in our study may have resulted in no OSw/oS cases in the wheat allergy group, the mechanism might not be relevant to the severity or localization of mast cells alone. The egg allergen might have a certain degree of specificity for inducing oropharyngeal symptoms, but details are unknown. Further large-scale studies are warranted to clarify the association mechanisms.

The present study has several limitations. First, since OSw/oS was determined by subjective symptoms, we cannot fully clarify whether it is an allergic symptom in the oral cavity and pharynx or exclude the possibility of oral discomfort being a psychological reaction to anxiety or refusal to ingest allergenic foods. Oropharyngeal discomfort can develop in various clinical settings and may also have a psychosomatic dimension [21]. Since patients enrolled in this study were in the pediatric age group, we cautiously determined the safe dose at which the allergen did not induce systemic reactions during the OFC, carefully observed the symptoms during the OFC, asked the patients and their guardians about the appearance of reactions and their subjective complaints in detail, and investigated the prevalence of OSw/oS. Although we believe that these countermeasures can minimize the number of indistinguishable non-OSw/oS cases, we cannot exclude the possibility of a wrong decision being made, since oropharyngeal symptoms in infants who could not verbalize their symptoms were assessed by nonspecific facial expressions and behaviors. Second, the guardians of patients with OSw/oS were instructed to reduce the dose of allergenic food or decrease the dose escalation rate. This may have prolonged the treatment period and reduced the likelihood of tolerance, while this may have allowed patients to continue OIT to achieve tolerance. Third, the exact effect of the intervention cannot be clarified due to the lack of control groups, and unmeasured confounding factors may remain since the present study was not a randomized trial. Lastly, subgroup analysis was difficult because of the limited number of patients for each food allergen.

In conclusion, we found that patients with food allergy, especially egg allergy, with OSw/oS achieved a lower rate of tolerance. Recognizing the high risk of intolerance associated with OSw/oS may help patients, their family members, and medical professionals prepare for the challenge of continuing OIT.

## Data Availability

The data that support the findings of this study are available from the corresponding author upon reasonable request.

## Abbreviations

OSw/oS: Oropharyngeal symptoms without systemic reactions
OFC: Oral food challenge test
OIT: Oral immunotherapy
HR: Hazard ratio
CI: Confidence intervals
